# Spontaneous mentalizing in patients with schizophrenia – a meta-analysis

**DOI:** 10.1192/j.eurpsy.2024.1552

**Published:** 2024-08-27

**Authors:** T. Csulak, R. Herold, G. Berke, Z. Sipos, K. Farkas, P. Hegyi, T. Tényi, A. Hajnal

**Affiliations:** ^1^Department of Psychiatry and Psychotherapy; ^2^Institute for Translational Medicine, University of Pécs, Pécs, Hungary

## Abstract

**Introduction:**

Mentalizing helps us to understand the behaviour of others in our everyday social interactions. Spontaneous mentalizing without explicit instructions refers to representing mental state attribution. Several studies have described social cognitive deficit in schizophrenia, which largely determines the functional outcome of the disease.

**Objectives:**

To better understand the involvement of spontaneous mentalizing in schizophrenia, we consider it important to summarize the results of studies that used indirect instruction to measure spontaneous mentalizing performance in schizophrenia.

**Methods:**

In our meta-analysis, we conducted a systematic search of four large databases (MEDLINE, EMBASE, Cochrane Central Register of Controlled Trials [CENTRAL], Web of Science). A total of 14 articles were involved.

**Results:**

Based on our findings, the performance of patients with schizophrenia is significantly weaker than in the average population for both scripts with mentalizing interactions (MD: -0.63; 95%CI (-0.90, -0.35); p=0.0021), and with goal-directed movements (SMD: -0.55; 95%CI (-0.97, -0.13); p=0.02). The intentionality of expressions used by patients with schizophrenia is significantly lower compared to the average population (for both animations with complex social interactions: MD: -0.99; 95% CI (-1.39, -0.59); p=0.0003; and with goal-directed movements: MD: -0.31; 95% CI (-0.53, -0.08); p=0.0218). We have found no significant difference neither in appropriateness nor in intentionality of verbal terms between the two goups in the case of animations with random movements.

**Conclusions:**

Based on the meta-analysis, we found poorer performance in schizophrenia in spontaneous mentalizing. We also found poorer performance in tasks with goal-directed movements used as control tasks, suggesting a more pervasive impairment of mentalizing in schizophrenia. These deficits may affect the functional outcome of the disease and could potentially have therapeutic implications.

**Disclosure of Interest:**

None Declared

